# P-2014. Online Clinician Education Curriculum Results in Performance Changes in Clinical Practice Related to Incorporating COVID-19 Antiviral Therapies for Non-Hospitalized Patients

**DOI:** 10.1093/ofid/ofae631.2171

**Published:** 2025-01-29

**Authors:** Arun S Nair, Maria B Uravich, Catherine Capparelli

**Affiliations:** Medscape Education, TARRYTOWN, New York; Medscape Education, TARRYTOWN, New York; Medscape Education, TARRYTOWN, New York

## Abstract

**Background:**

This study examined whether online continuing medical education (CME/CE) focused on evidence-based best practices for incorporating COVID-19 antiviral therapies in non-hospitalized patients would result in improved identification of high-risk patient groups and the adoption of new clinical practices.

**Methods:**

Clinicians who participated across a curriculum of 3, live and online, 30-minute CME/CE video discussions between experts. Performance was assessed by inviting learners to complete a survey 30-60 days post-education, identifying practice changes and the degree to which clinicians experience barriers to those changes. The data was collected from 11/29/2022 to 06/21/2023.

**Results:**

There were 232 learners consisting of PCPs, Emergency Medicine Physicians, ID Specialists who completed the survey. The analyses from the 3 programs showed that approximately 98% of learners made a practice change or had practices reinforced due to education. Five key practice changes that were modified/implemented as a result of the education included:

- Identifying patients at higher risk for developing severe COVID-19-related complications (69%)
- Incorporating new information about treatment options for non-hospitalized patients with COVID-19 (67%)
- Ensure patients receive treatment within the appropriate therapeutic window (60%)
- Educating patients on COVID-19 and available antiviral treatments (58%)
- Screening patients for potential DDIs prior to COVID-19 antiviral initiation (55%)
- Some of the barriers to implementing these practice changes include:
- Patients present outside the recommended treatment window
- Unfamiliar with potential drug-drug interactions for COVID-19 antivirals
- Patient concerns or questions regarding available antivirals
- Unfamiliar with guideline recommendations for available antivirals
- Lack of time during office visits to educate patients on the importance of timely testing and treatment

**Conclusion:**

The practice changes identified in this assessment provide compelling evidence that participation in online CME/CE prompts adoption of changes in practice related to COVID-19 antiviral therapies in non-hospitalized patients.

Acknowledgement: This education was supported by an independent educational grant from Pfizer Inc. and Merck & Co, Inc.

**Disclosures:**

All Authors: No reported disclosures

